# Drivers of sustainability transformations: leverage points, contexts and conjunctures

**DOI:** 10.1007/s11625-021-00957-4

**Published:** 2021-04-27

**Authors:** Björn-Ola Linnér, Victoria Wibeck

**Affiliations:** Department of Thematic Studies-Environmental Change, Linköping University, Linköping, Sweden

## Abstract

While increasing hopes are being attached to deliberate societal transformative change to achieve the targets of the 2030 Agenda and the Paris Agreement, questions remain about whether and whereby such profound systemic change can be governed. This paper analyses *how* transformative changes are intended to be encouraged and achieved, *where* and *when*. The paper explores critical drivers and how they relate to leverage points at different places in the societal systems. The paper builds on a comprehensive sense-making analysis of scholarly literature, policy documents, including countries’ contributions to the Paris Agreement and national reviews of progress towards the UN Sustainable Development Goals, international news media and lay focus group discussions on five continents. There are great variations in how drivers were made sense of in the data. The many ongoing interacting transformations across societies involve different social, cultural, and political contexts, while the implementation of the 2030 Agenda also contains goal conflicts and unavoidable trade-offs. The paper highlights four categories of drivers as particularly important to consider in view of international transformation efforts: technological innovations, political economy redistribution, new narratives, and transformative learning. Four features are important for bringing clarity on how deliberate transformations can be encouraged: (1) the function of drivers in enabling and restricting transformations of societal systems characterised by detailed or dynamic complexity, (2) cultural and geographical contexts of transformations, (3) where in the systems the drivers are intended to intervene, and (4) the role of critical junctions in time, where transformative trajectories can branch out.

**Supplementary Information:**

The online version contains supplementary material available at 10.1007/s11625-021-00957-4.

## Introduction

Due to the very slow, or even negative, progress towards the Paris Agreement’s temperature goals and long-term climate neutrality, while also delivering the Sustainable Development Goals, governments, civil society actors, and a growing number of scientists are turning their attention towards the concept of societal transformations. They are charting a break with fossil-based, resource-wasting economies towards achieving sustainable, decarbonised, and climate-resilient ones. Societal transformation implies fundamental, systemic, non-linear changes (Brand 2016; Fazey et al. 2018; Hölscher et al. 2018; Linnér and Wibeck 2020; Patterson et al. 2017).

While there appears to be mounting agreement that the planetary crises and the sustainability goals require transformative changes (e.g., European Commission 2019; IPCC 2018; IPBES 2019), there is far from being a consensus on how such changes can be initiated. Yet, we can see some reoccurring drivers when actors with varied geographic and cultural background make sense of how societal transformations can be achieved. The aim of this paper is to analyse how actors from diverse contexts around the world understand whereby transformative changes towards sustainability can be instigated and encouraged. In doing so, the paper explores what drivers are identified as critical and how they can influence transformations.

## Conditions for societal transformations

Societies are complex systems. It is a formidable task to disentangle the extent to which the feedback loops of radical and comprehensive societal changes are the product of efforts to guide, manage, and govern the changes and the extent to which they are the outcome of the emergence of inadvertent transformations within other parts of society.

A key conclusion in complex system theory is that long-term intentional schemes seldom evolve as planned, because it is essentially impossible to anticipate all the irregular, non-linear interactions, reconstitutions, and feedback loops between a multitude of elements and factors evolving over time as well as the interplay between systems and their environments. Thus, we find it helpful to distinguish between the transformation of systems that are characterised by *dynamic complexity* and those that are characterised by *detailed complexity*. Human societies are an epitome of adaptive complex social systems in which the ‘effects over time of interrelatedness are subtle and the results of actions are not obvious; or where short-term and long-term effects are significantly different; or where effects locally are different from effects on a wider scale’ (Flood 2002, pp. 13–14; cf. Senge 1990, Miller and Page 2007). More  demarcated systems can instead demonstrate detailed complexity, ‘in which there are many variables that may be difficult, but not impossible, to consider as a whole’ (Flood 2002, p. 13). Examples of such more comprehensible system changes could exist within certain energy, agricultural or urban sectors.

Considering the grand challenge of instigating a profound, enduring, and non-linear change in complex systems, the literature grapples with how this can be done effectively. There is little consensus in the growing literature on transformational governance about how change can be encouraged. Neither is there any coherence on the key concepts to characterise societal system change. For example, Sachs et al. (2019) identify four general governance mechanisms for implementing intended transformations to sustainability. Petra Kuenkel’s (2019) *Stewarding Sustainability Transformations* uses the concept of drivers, while The Independent Group of Scientists (2019) appointed by the United Nations’ Secretary-General and the IPBES report (2019, see also Chan et al 2020) instead use the concept of a lever (Table 1).

**Table 1:** Examples of proposed change mechanisms to achieve deliberate transformations

Governance mechanisms (Sachs et al. 2019)	Drivers (Kuenkel 2019)	Levers (The Independent Group of Scientists 2019)	Levers (IPBES 2019)
Goal-based design and technology missions	Innovation	Science and technology	Incentives and capacity building
Social activism to change norms and behaviours	Narratives	Individual and collective action	Coordination across sectors and jurisdictions
Goal-based organization of government and financing	Governance	Governance	Adaptive decision-making
Diplomacy and international cooperation for peace, finance and partnership	Regulations	Economy and finance	Environmental law and implementation
	Metrics		Pre-emptive action
	Structures		

The order is not hierarchical and has been adjusted to facilitate comparison

These key concepts all draw on metaphors, which is a powerful resource for making sense of the world. Metaphors not only frame our understanding, but can ultimately affect how we engage and act in societal affairs (Lakoff and Johnson 1980). For that reason, we would caution against using the metaphorical concept of a ‘lever’ for complex non-linear societal change as it ‘suggests a simple system where one change can result in a desired outcome’ (Skoog and Bilica 2002, p. 445). This connotation is often enforced by a figure showing a lever elevating or setting a transformation in movement (IPBES 2019:40). A straightforward, direct, cause-and-effect logic will not suffice to comprehend the complex interactions, feedbacks and irregularities of societal transformations.

The metaphor of *leverage points*, introduced by Donella Meadows (2008), does not necessarily convey the same connotation. The concept instead serves to identify ‘places to intervene in a system where a small change could lead to a large shift in behavior’ (Meadows 2008, p. 145). By focusing on the different features of a system, we are encouraged to consider not only what compels systems to change, but also the effects that different drivers may have within the various parts of a system. Meadows cautions against using her analysis as ‘a recipe for finding leverage points. Rather, it is an invitation to think more broadly about system change’ (Meadows 2008, p. 147).

Meadows discusses the leveraging of change in any system, which she defines as ‘a set of things—people, cells, molecules, or whatever—interconnected in such a way that they produce their own pattern of behaviour over time’ (Meadows 2008, p. 2). A system could be, for instance, an economic sector, a societal sector, such as energy or agriculture, a business organisation, a city, an organism, or an ecosystem. Meadows organised the leverage points in order from least influential to most important for changing a system. She identified twelve leverage points (Meadows 2008, pp. 147–165) (Table 2).

**Table 2:** Donella Meadows’ leverage points for intervening in a system (Meadows 2008)

Order of influence	Place to intervene in a system
12	Constants, parameters, numbers (such as subsidies, taxes, standards)
11	The sizes of buffers and other stabilising stocks, relative to their flows
10	The structure of material stocks and flows (such as transport networks, population age structures)
9	The lengths of delays, relative to the rate of system change
8	The strength of negative feedback loops, relative to the impacts they are trying to correct against
7	The gain around driving positive feedback loops
6	The structure of information flows (who does and does not have access to information)
5	The rules of the system (such as incentives, punishments, constraints)
4	The power to add, change, evolve, or self-organise the system structure
3	The goals of the system
2	The mindset or paradigm out of which the system—its goals, structure, rules, delays, parameters—arises
1	The power to transcend paradigms

When looking more closely into the mechanism for change in Table 1, technological innovations are mostly seen as influencing (9) The lengths of delays, relative to the rate of system change and (8) The strength of negative feedback loops, relative to the impacts they are trying to correct against. Governance mostly intervenes in (4) The power to add, change, evolve, or self-organise system structure; and (3) The goals of the system. Narratives and social activism to change norms and behaviour primarily address (2) The mindset or paradigm out of which the system arises. Meadows’ primary way to intervene, (1) The power to transcend paradigms, is less pronounced in the change mechanisms listed in Table 1.

Given that various concepts are used in the scholarly literature to address how transformations can be achieved, and some concepts are sometimes left undefined and sometimes used in different ways, we propose the following terminology for analysing deliberate efforts to transform complex societal systems (Table 3).

**Table 3:** Concepts used for describing how transformational change occurs

Change concept	Definition
Drivers	Factors that compel a system to change
Governance mechanisms	The pathway or process by which a targeted outcome is achieved
Intervention	A particular element of policies, programmes, or strategies that is designed to produce a situation which can secure desired outcomes
Leverage points	The part of the system that can be influenced for a proportionally greater effect on the whole system

We use *drivers* for the broader factors that compel a system to change. *Governance mechanisms,* defined as ‘the pathway or process by which a targeted outcome is achieved’ (Linnér and Wibeck 2019, p. 164), can be used to operationalise drivers. We use this for the empirically identified specific intentional societal processes or policy pathways that are believed to be critical for transformational change. An *intervention* is ‘a specification of what must be done to achieve the desired goals’ (Chen 1990:40). It is generally more specific than the governance mechanisms because it refers to a particular element of policies, programmes, or strategies that is designed to produce a situation which can achieve desired outcomes (Chen 1990; Mickwitz 2003). *Leverage points* specifically address what part of the system that is influenced for a proportionally greater effect on the whole system.

## Materials and methods

This paper builds on mixed-methods and cross-country studies of how societal transformations are made sense of around the world in different fora. The studies analysed differences as well as commonalities across communicative genres, different geographies and actor groups. For this purpose, we conducted a comprehensive sense-making analysis (Wibeck and Linnér 2021). We used multi-strand analyses (Bryman 2006) that pooled different types of methods analysing four types of data: (1) Research literature; (2) Policy documents, including (a) all Intended and Decided Nationally Determined Contributions (NDCs) to the Paris Agreement and Voluntary National Reviews (VNRs) of the UN Sustainable Development Goals submitted by 2018, (b) the UN 2030 Agenda and background reports, (c) the European Green Deal and background reports; (3) International, English-language media texts published between 2007 and 2016 and identified through the Retriever data base; (4) Focus group discussions.

The first three types of data were analysed to generate a broader overview of how sense-making varies across the different genres and countries over time. To be able to analyse the sense-making process in action, together with locally based colleagues, we conducted focus group interviews with citizens in five countries in five continents: Cabo Verde, China, Fiji, the USA and Sweden. In each location, four focus groups were conducted, encompassing a total of 136 participants. Since we aimed for a diverse yet targeted sample of sites where transformation is high on the policy agenda, we selected culturally, politically, and economically diverse sites with different environmental and social challenges, where the concept of transformation has been explicitly grappled with in official policy documents (see Wibeck et al. 2019).

First, we analysed each dataset in turn, performing so-called vertical analyses (Wibeck and Linnér 2021). Based on our initial reading and our analysis of the societal transformations literature, we developed an analytical template. This was used to identify how the sources made sense of transformations in terms of goals, scale, scope, pace, drivers, agents, and periodisations of transformative change. In the vertical analyses, we also focused on mapping sense-making strategies, such as framing, metaphors, analogies, distinctions, and stories.

These vertical analyses were used as a basis for approaching the data sets horizontally, where we identified varieties and commonalities across our different empirical sources. Here, we particularly sought to identify overarching societal narratives of transformations that recurred in the datasets. For more details on the data and methods of these studies, see Electronic Supplementary Material, as well as Linnér and Wibeck (2019), Wibeck et al. (2019) and Wibeck and Linnér (2021). In this paper, we focus on the analysis of how drivers of transformation were discussed in the different data sets.

## Results

### Drivers of change

The scholarly literature on societal transformations to sustainability is predominantly concerned with governance for transformations. It seeks to identify conditions for leveraging large-scale systematic changes, whether it is in specific sectors, socio-technical–ecological systems, or entire cultures (e.g., Göpel 2016; Kuenkel 2019; Olsson et al. 2010; Patterson et al. 2017; Scoones et al. 2015).

A striking result emerging from our mapping is that, in many places across the world, people are equally concerned with the ongoing transformations that they are struggling to cope with. How ongoing and emergent transformations affect sustainability governance efforts is illustrated by the European Commission’s analysis of a new long-term climate strategy for 2050:

Deep decarbonisation will not be the only transformative trend that will affect the EU and global economy over the coming decades. For example, the transformation will take place in a context of an ageing EU population and evolving globalisation as well as some effects of climate change (much more moderate though if decarbonisation objectives are achieved).

(European Commission 2018, p. 217)

In particular in the low- and middle-income countries and in vulnerable communities, the reality of coping with ongoing transformations was most pronounced. Most of the 50 low- and middle-income countries’ NDCs and their background documents that referred to transformations build on-ongoing policies of economic and social transformation. These do not emanate primarily from the sustainable development discourse, but from post-colonial experiences and long-standing conclusions within development economics or human development theory. For example, in Cabo Verde, individuals, civil society and politicians all pursue efforts to cope with their vulnerability not only to climate change, but also to evolving global economic power relations and geopolitical developments as well as attempting to capture potential prospects for the islands in the South Atlantic Ocean (Linnér and Wibeck 2019; Wibeck et al. 2019).

These are examples of governance of ongoing transformations. That is, the governance efforts seek to capture drivers that can make the societies robust enough to cope with ongoing long-term change under dynamic complexity. For instance, in Fiji, education is seen as a driver that will increase awareness, competence, and agility to manoeuvre within a transforming Pacific characterised by marginality in global economic exchange and geopolitical exposure.

For the deliberate transformations, our data displayed a broad set of drivers, ranging from technological innovations and economic incentives, to educational efforts and shifts in mindsets.

In our data, visions, values, political leadership, public engagement, and communication were generally identified as important in providing a direction for deliberate transformations that address the goals and paradigms of the system. Other drivers sought to enable transformative change. These included political decisions, institutional change, rules and practices for economic exchange, technological innovations and deployment that, in Meadow’s (2008) terms, mostly target the strengthening of negative feedback loops. Education, shifts in perspective, and lifestyle changes rather seek to empower people to change system structures and paradigm.

Lifestyle changes were often presented—especially in our media material—as a second-order enabler. That is, they were usually not seen as the primary driver of change. Although lifestyle changes were not redundant, they were themselves enabled by necessary interventions through, e.g., political decision-making, or awareness-raising in media and education. Shifts in perspective can similarly be seen as second-order enablers, coming about as a result of factors, such as transformative learning or new narratives.

In the major societal transformations spanning across societies that are needed to achieve the 2030 Agenda, we see four drivers as necessary, even if they may not be sufficient or the only drivers of importance: technology innovations, political economy redistribution, new narratives, and transformative learning. The four drivers were accentuated across our data and to some extent highlighted in previous literature (see Sect. 2). They intervene in different places in the societal systems and have particular implications for various actors in particular contexts, with dissimilar political priorities and societal visions. They illustrate the breadth of necessary societal drivers and how they may be linked.

#### Technology innovations

The international policy documents and media reports foregrounded innovation and diffusion of renewable and resource-efficient technologies as critical interventions not only for energy production and consumption, but also for the transformations of entire societies, even on a global scale. In the focus groups as well, participants expressed their hopes in new low-carbon technologies, although at times they also cautioned against investing too highly in hopes for the development of future technologies at the expense of individual and collective engagement. In the NDCs and VNRs, technology innovation was the most common transformational driver. Taken together, our empirical sources paint a picture of the broad-based development of technologies, requiring investments in research, development, demonstration and deployment, infrastructure, support and training, legislation and regulation to nurture not only technological, but also social innovations.

The Intergovernmental Panel on Climate Change makes the same assumptions. Its Fifth Assessment Report established that ‘the use of improved and new technologies is an inherent element of society’s transformation’ (Clarke et al. 2014, p. 466). The special report on limiting global warming to 1.5 °C maintained ‘with high confidence’ that the ‘system transitions’ required to achieve this temperature target include the ‘widespread adoption of new and possibly disruptive technologies and practices and enhanced climate-driven innovation’ (IPCC 2018, p. 24).

When the attention is only on reducing emissions, a sole focus on technological fixes may seem sufficient. But, as numerous studies have shown, this is not the case for any major societal transformation (e.g. Linnér and Wibeck 2019; Pielke and Linnér 2019; Sachs et al 2019; TWI2050 2018). Even more so, when other sustainable development goals, such as poverty eradication, are included in the aspirations, then social aspects, such as behavioural change, need to be included to achieve the same level of confidence in the role of technology as a driver of societal transformations (IPCC 2018).

To what degree do transformative changes pivot on technological advancements? There is an enduring discussion about whether we have the necessary technological capacity to remain below the 450 ppm CO_2_ equivalent level that is assessed to be required to keep the world below the 2 °C target (Clarke et al. 2014; Pielke 2010). The assessment depends, of course, on how we anticipate that societies will develop, and on how the transformations unfurl. The focus groups’ views on technology extended from holding great promise to being inherently environmentally risky. The role of technology was essentially tied to their visions of what a sustainable society entails (Wibeck et al 2019).

What technological innovations are required as drivers of societal transformations depends on what kind of world we want. If we anticipate little public or political support for degrowth or low economic growth, profound decarbonisation will be unintelligible without the radically boosted deployment and proliferation of new energy technologies (Pielke 2010). But, if we anticipate other needs and priorities to also emerge as perspectives change, the dependency on disruptive technological innovation is very likely to decrease (Clarke et al. 2014).

#### Political economy redistributions

When international sustainable development is addressed, the distribution of wealth and economic power surface repeatedly as desired drivers in our focus groups, as well as in a majority of the NDCs and VNRs that deal with transformations. This is hardly surprising. Political economy distribution goes to the very heart of international sustainability-making since the dawn of international environmental cooperation in the 1960s (Linnér and Selin 2013).

The political economy aspects address the connections between the state and the institutions regulating economic change and how resources—whether monetary, material or non-material—are used and distributed within and between societies (Caporaso and Levine 1992; Gilpin 2016). In our data, we see the emergence of three lines of reasoning on political economy changes as critical drivers, either on their own or in tandem.

1. *Enhancing adaptive capacity to cope with structural changes*. Focus group discussions in Fiji and Cabo Verde as well as several NDCs and VNRs from low- and middle-income countries grapple with how to ease the negative social and economic effects of ongoing transformations. Economic redistribution, inclusive decision-making and enhanced access to and quality of education are the three primary governance mechanisms suggested to this end. For example, heightening public education levels or diversifying the country’s economic sectors are two key strategies for Cabo Verde to forestall the emerging transformations in the global political economy by enhancing its economic and social resilience. Here, the power to change the structure of the system is the focus. The Gambia’s NDC, referring to its *2020 Vision,* underscores that social transformation propelled by economic development inevitably generates ‘imbalances’ among social groups, as well as with the environment (Gambia 2015). The country’s sustainable transformation thus requires concerted, tolerant, equitable, and supportive societal structures that ensure a just distribution of resources as well as good governance to avert the ensuing negative side effects of transformation policies.
2. *Ensuring the legitimacy and effectiveness of transformative actions.* Societal transformations inevitably generate winners and losers in relative terms, both within and across countries. Just as power can be redistributed to new groups, so can societal transformations also entrench or reinforce social power structures at the expense of the already societally or ecologically vulnerable (Blythe et al. 2018; Stirling 2014). The significance of addressing distributional aspects to ensure the legitimacy of transformative actions in fossil-fuel-driven economies is attracting growing attention among for instance labour unions and the EU Commission.
  Here, the rules of the system are the main focus. For example, in the *European Green Deal*, the European Commission envisions an economic restructuring built on the principles of a circular economy and a just transformation that should ensure that regions with heavily carbon dependent sectors receive support to restructure the economy (European Commission 2019). Nevertheless, it is still an economy based on international competition and economic growth that avoids the issue of global redistribution. ‘It is a new growth strategy that aims to transform the EU into a fair and prosperous society, with a modern, resource-efficient and competitive economy where there are no net emissions of greenhouse gases’ (European Commission 2019, p. 2).
3. *Taking on the political economy power structures.* In the words of Andy Stirling (2014, p. 84), ‘Power is necessary for transformation, but this may be subverted if power itself is not transformed’. Just as technology permeates societies, so do the institutions for economic change. A fundamental transformation of an entire society needs to also involve a transformation of the political economy. That is not to say that it necessarily involves economic redistribution between the have and the have-nots of today. In our datasets, the envisioned actions range from governance mechanisms for economic restructuring of the global economy (e.g. Bolivia 2015; Ecuador 2015) to interventions such as the swapping of taxes to create new circular-economy incentives within liberal capitalism to adjustments of the cost of transformations to decarbonisation (e.g. Germany 2017).
  This third aspect of political economy is inherently politically contested. This may explain why it is only discussed in very generic terms in publications trying to find common grounds for transformative actions (e.g. Sachs et al. 2019; The Independent Group of Scientists 2019; UN 2015).

In the media texts, the political economy redistribution as a driver of sustainability transformations is seldom stand-alone. For example, one article dealing with poverty alleviation and social segregation in Haiti states that foreign financial support is essential for achieving the SDGs. Yet, this does not suffice on its own, it needs to be addressed in tandem with interventions to enhance education and public participation (Cision PR Web 2016).

#### New narratives

The importance of new stories or narratives for leveraging transformation is increasingly gaining recognition in the scholarly literature. Such stories are needed to break with current dominant narratives of humanity’s separation from nature and of the dominant economic growth paradigm that is dependent on cheap fossil energy and abundant natural resources (e.g. Kuenkel 2019; Leggewie and Messner 2012; O’Brien et al. 2019).

Four functions of narratives make them a transformational driver. (1) They can spur perceptions of possible and desirable societies. O’Brien et al. (2019) argue that stories are crucial in opening up people’s imagination and actualize ‘not-yet-here’ possibilities, as well as in questioning taken-for-granted assumptions. How we imagine what future worlds may be like, and how we deliberate on them in ethical and political terms, lays the foundation for political behavior and decision-making (Milkoreit 2016; Veland et al. 2019). (2) They can engage the personal sphere in a collective story. Kuenkel (2019) emphasises the role of emotionally engaging and ‘enlivening’ narratives that can contribute to revitalize ‘the human capacity to shape a sustainable future collectively at scale’ (p. 225). (3) The spread of new narratives can influence systems of meaning that might in their turn have an impact on priorities and guide new types of actions. For example, Riedy et al. (2019) argue that although the fossil fuel divestment movement so far has had a limited impact on fossil fuel companies’ actual market value or the conditions for carrying out their businesses, it has contributed to establishing a new narrative that might influence factors such as the future investment policies of finance institutions. In this sense, narratives can be conceptualised not only as an intervention that changes policy practices, but ultimately intervenes on the paradigmatic, the sense-making, level. Toppling a persuasive, dominant, narrative that posits carbon-driven economic growth as the bedrock for societal wellbeing by one emphasising renewability, ecological consciousness, and social relations, would powerfully intervene to make fundamental changes throughout societies, even across civilisations, desirable, achievable, and well-founded.

Examples of how new narratives are foregrounded as a key driver for transformative change can also be found in a handful of the policy documents and some of the focus groups included in our analysis. For instance, in half of the US focus groups, some participants highlighted the importance of envisioning desirable futures to spur change. Similarly, in reviewing its Agenda 2030 efforts, The Swiss Federal Council argued that ‘the SDGs are embedded in a narrative of transformative change, which is needed to realise a common vision of ending poverty in all its forms and of promoting social inclusion and universal human development that respects human dignity, human rights, and planetary boundaries’ (Switzerland 2016, p. 12).

The more comprehensive narratives on societal transformations that we found in the policy documents and media texts often stemmed from the Global South. In the focus groups, transformation narratives at times drew on analogies with previous deliberate transformations which were seen as inspirational examples of how change can occur. Examples included Cabo Verde’s change from a least-developed to a middle-income country, or women’s rights issues.

Five core narratives, characterised through the core metaphors around which they were organised, recurred across the data: transformation as a journey, a building process, a war, co-creation, and recuperation (for an in-depth analysis of each of these narratives, see Linnér and Wibeck 2019, ch. 6). Each of these metaphorical ways of comprehending transformation processes has different advantages and limitations for stimulating engagement, and they may speak to different audiences, supplying a bouquet of alternative or integral models for societal transformations.

While the journey narrative risks downplaying ongoing transformations through its future-oriented focus and eclipsing transformation goals by overstating the journey itself at the expense of where to arrive (Milne et al. 2006), its focus on the journey also connotes that striving for just and sustainable societies need to be ongoing. The building narrative emphasises joint endeavous to achieve long-term objectives (Charteris-Black 2004; Koteyko and Ryazanova-Clarke 2009) but, like the journey narrative, it can also obscure existing capacities and previous achievements (Hagelsteen and Becker 2013; Walters 2007). While the war narrative is conflict-oriented (Carew and Mitchell 2006; Princen 2010; Romaine 1996) and may de-emphasise collaborative efforts and legitimise authoritarian decision-making, it can also allude to ideas of mobilisation, endurance and sacrifice for the common good (Anshelm and Hultman 2015; Cohen 2011). The co-creation narrative signals collaboration, dialogue and participation by multiple actors in transformation, but does not provide clarity about the procedural challenges of engaging and orchestrating a wide variety of actors in deliberative activities. Lastly, the recuperation narrative presents notions of restoring what was lost, either in societies or from an environmental perspective.

What these narratives have in common is that they represent transformations as processes rather than goals and all assume deliberate transformations, presuming that some level of successful interventions is not only necessary, but possible.

As one of the functions of narratives is to make chains of event intelligible, they may contribute to downplaying the complexity of systemic transformative change. However, narratives can also be instrumental for sense-making because they draw on metaphors and analogies to bring shape to complex relationships. They can empower people not only to change the goals and the paradigm, but also to make sense of system relationships and orient themselves within a dynamic complexity.

#### Transformative learning

*Perspective transformation* (Mezirow 1991)—is accentuated across our data as a key transformative feature. It entails revisiting taken-for-granted assumptions, world views and behaviours. For such shifts to occur, not only new narratives but also transformative learning is needed (Crompton 2011; Mezirow 2000; O’Brien and Sygna 2013). According to pedagogic theories on transformative learning (e.g. Boström et al. 2018; Howie and Bagnall 2013; Mezirow 2000), perspective transformation may come about as a result of a disorienting dilemma, such as a disruptive event—that is, a predicament that is confusing enough to warrant epistemological change, through which ‘we change the very form by which we make meanings’ (Boström et al. 2018, p. 6). Such a disorienting dilemma could be addressed through deliberate analytical processes that encourage consideration of assumptions that people are often unaware they are making, as well as through intentional actions that can support reflection upon and rethinking of social worlds.

Transformative learning may provide tools to grapple with uncertainties in how the long-term deliberate planning of complex systems will unfold. As illustrated in some of the policy documents and media texts, education and learning are needed to encourage a shift towards a ‘culture of sustainability’ (Italy 2017), facilitate the capacity to manage risk and strengthen resilience building (Dominica 2015) or to enable people to become environmental stewards working towards sustainability (The Philippine Daily Inquirer 2008).

A recurring topic in all the places included in our focus group study, and in half of the analysed policy documents from many low- and middle-income countries, is that learning, in a broad sense, provides necessary empowerment. Educational initiatives range from public information campaigns enhancing knowledge about environmental changes to initiatives that advance school systems to raise the general level of education. These are seen as essential not only for creating legitimacy and empowerment for sustainability transformations, but also for providing agency and adaptive capacity to manoeuvre within an ever-transforming world, not only to be able to change the system structure, but ultimately to have the power to transcend paradigms.

## Discussion

Many different visions and goals, interests, and geographical contexts were reflected in our data, and we saw great variations in how drivers were made sense of. Consequently, there is no single blueprint for all pursuits of sustainability transformations (Linnér and Wibeck 2019). First, the drivers naturally hinge on how the ultimate sustainability goals are prioritised. Even if we let sustainability be defined by the 2030 Agenda, it is filled with goal conflicts and unavoidable trade-offs (Bowen et al. 2017; Nilsson and Wietz 2019; OECD 2018), which will affect how interventions, governance mechanisms, and ultimately drivers are prioritised. Second, the many ongoing interacting transformations across societies involve various social, cultural, and political contexts.

Similarly, there is little consensus in the recent literature on how key concepts for achieving deliberate transformative change, such as levers, governance mechanisms, interventions and drivers, should be defined and what actions they need to include. One reason for this may be that the studies draw on different cases. And as all transformations are context-dependent, it is not likely that they could be reached by a universal set of interventions. Many papers also take a broad perspective on transformation to reach global sustainable development goals and thus tend to outline rather generic change factors. Thus, we suggest that the next phase of transformation research should explore the many variations and nuances in the multitude of transformations that are pursued globally.

Around the world, many people are already making efforts to address ongoing transformations, although this tends not to be accentuated in the discourse on transformations towards sustainability. Yet, for those who are economically, socially and ecologically vulnerable, struggling to cope with emerging transformations, it is evident that societal transformations are associated with dynamic complexity, where the transformative intentions encounter not only irregularities and feedbacks, but existing power structures, unfolding socio-economic processes, socio-technical feedbacks and geopolitical struggles.

In a world where societal systems need to be fundamentally changed, where we all will be treading unknown paths, lessons can be learned from experiences of navigating under uncertainty and confined control in the midst of emerging transformations. For instance, transformative learning exercises could take inspiration from dialogue-oriented processes, such as the *talanoa* sessions that formed part of the reconciliation processes, charting a way towards a different society, after the coup in Fiji in 2000 (Robinson and Robinson 2005), or the co-creation exercises for post-war planning in Colombia and post-apartheid planning in South Africa (Kahane 2012). While these exercises were formed by, as well as designed to match, specific cultural circumstances, there is much to learn from what they have in common: acknowledging that the present situation is intolerable and that change is imperative, and emphasising the need to listen to and learn from one another's experiences, while also bringing up painful issues rooted in the conflicts that originally motivated the interventions.

When considering the governance of sustainability transformations, we need to ask how different factors can either enable or restrict societal transformations of systems that are characterised by detailed or dynamic complexity. The transformation of an entire society is inherently dynamic in its complexity, and actors will encounter and must adapt to and learn from evolving interactions and feedback loops (Flood 2002; Miller and Page 2007), while the complexity of a transport or energy system can be detailed. Whereas, typically, technologies can respond to detailed complexity, education is ideally designed to provide agile skills to respond to uncertainties, reflexivity, intricacy and feed-back loops. However, what determines how the drivers function to respond to detailed versus dynamic complexity is rather how they are harnessed through specific governance mechanisms and interventions at various places in the system.

The distinction between dynamic and detailed complexity can be illustrated by contemplating the five core narratives of transformation processes identified through our analysis. Some narratives take their point of departure in dynamic metaphors, such as the co-creation of knowledge and action between a multitude of stakeholders, or as a recuperation process through which the planet or society can be restored. These narratives are well positioned to respond to societies’ dynamic complexity. In contrast, conceiving of transformation in terms of a journey, a building process, or a war may convey an understanding of societies as characterised by detailed complexity, if these narratives express a direct causality, or a linear chain of events.

A key conclusion is that none of the four drivers discussed above suffices on its own. Rather, combinations of drivers are needed to support transformations of such complex systems as societies across the world. Also, multiple policy instruments are needed to intervene in different parts of these systems (Dentoni et al. 2017; Linnér and Wibeck 2019; Patterson et al. 2017). Similarly, through the concept of ‘radical incremental transformation’, Maja Göpel (2016, p.7) outlines how new economic paradigms can emerge out of experimentation, social innovations, and learning; for example, in the Transition Town movement. Changes in political economy can underpin the paradigmatic shifts that, in turn are fundamental for attaining the mind shifts necessary for societal transformations to sustainability.

The four drivers intervene at different places in systems, in the language of Meadows (2008). For example, technological innovations can affect the size of buffers and the power to change the system structure. Technology as a driver also concerns the lengths of delays and rate of system changes because technological breakthroughs can rapidly change the conditions and flows within a societal system. However, the innovation chain can also prove to be an uncertain and prolonged intervention when a rapid system change is desired. New narratives, along with changes in political economy, can contribute to guiding mind-shifts needed for transformative change. Transformative learning is a particularly important driver for transformations of dynamic complex systems, since such learning can bring about shifts in perspective that, in the long run, can enable awareness to not only shift paradigms and mindsets, but ultimately empower people to transcend paradigms.

For deliberate sustainability transformations a third factor, besides drivers and places to intervene in the system, is whether there are critical junctures where a radical break with prevalent trajectories has a greater chance to succeed. In analyses of socio-technical path dependencies and historical institutionalism, periods of stability are punctuated by critical junctures, that is, occasions that can lead to profound societal change, where key features of societies move onto new paths (Capoccia and Kelemen 2007; Levin et al 2007). In particular, conjunctions can open possibilities for rapid changes of prevalent structures, for example, when a disorienting dilemma temporally intersects with social or technical innovations, and economic restructuring (Mahoney 2000; Skocpol 1979).

The COVID-19 crisis has brought the role of disruptors for sustainability transformations to the fore. Disruptors refer to occurrences that interrupt a system or a process from continuing as usual or as expected. Its Latin root means to break apart. A disruptor can be a technology, a political event, an environmental disaster, or a virus. In times of crisis, social structures and institutions are put to the test. Normal practices and habits are called into question. Disruptions can uproot and alter how we make sense of our world, how we behave, do business, do politics, learn, and go about our daily lives. In *Logics of History*, Sewell Jr (2005) elaborates on theories of how structural change and social transformation can occur:

In times of structural dislocation, ordinary routines of social life are open to doubt, the sanctions of existing power relations are uncertain or suspended, and new possibilities are thinkable. In ordinary times, cultural schemas, arrays of resources and modes of power are bound into self-reproducing streams of structured social action. But in times of dislocation … resources are up for grabs, cultural logics are elaborated more freely and applied to new circumstances, and modes of power are extended to unforeseen social fields. (Sewell Jr 2005, pp. 250–251).

The underlying assumption behind the disruption-conditional transformations is that transformations require some kind of ‘quantum leap’ to be able to break away from path dependencies, or create legitimacy for radical societal change. These disruptors create windows of opportunity for new experimentation, perceptions, priorities, and social and technological innovations to develop and flourish. Ultimately, they create an opportunity to transcend our preconceived notions about the goals and interactions of societal systems.

Further research is needed into the role of critical junctures and into the extent to which disruptive events affect the functioning of the drivers and how they can be harnessed through interventions—for instance, leading to new narratives and perspective shifts—or whether disruptions rather amplify perspective shifts that are already underway.

## Conclusion

The paper highlights four features that are important for bringing clarity on how deliberate transformations can be encouraged: (1) the function of drivers in enabling and restricting transformations of societal systems characterised by detailed or dynamic complexity, and through which specific governance mechanisms and interventions are operationalised, (2) the cultural and geographical contexts of transformations, (3) where in the systems the drivers are intended to intervene, and (4) the role of critical junctions in time, where transformative trajectories can branch out.

The mission to foster transformative change in the dynamic complexity of societies is even more daunting, as the 2030 Agenda sets out to do this in a concerted effort throughout the world. It underscores the importance of reflexivity, examples, agility, and conjunctions.

First, for the implementation of transformational strategies, we need to move away from a too-generic identification of drivers. Analysts and policymakers need to recognise the great variations in how drivers are made sense of due to the many ongoing interacting transformations across societies, which involve various social, cultural, and political contexts. The implementation of the 2030 Agenda also contains goal conflicts and unavoidable trade-offs.

Second, changes in systems of detailed complexity, such as bounded urban or transport systems, can provide tangible and appreciable outcomes. Thus, they do not only create conditions for change, but they also provide examples to inspire dialogues, creativity, and reflections. Previous literature (e.g., Folke et al. 2010; Geels and Schot 2010) has highlighted the importance of niche developments for creating space for innovations and providing the impetus for transformational change by, for example, spurring learning, enhancing processes and technologies, developing institutional frameworks, and creating public, political, and producer support.

Third, this must be done at the same time as we also intervene to foster agility and capacity to respond to the dynamic complexity of society-wide transformations evolving over time. The technologies, institutions, or economic measures that we need are subject to the narratives and deliberations about what kind of society we want. So, while the niche developments are important to provide impetus, transformative learning and co-creative dialogues are fundamental drivers for these niche transformations to inspire society-wide system change marked by irregular, non-linear, and feedback interactions.

Fourth, we also need to consider how the drivers can intervene effectively in societal systems, and how critical junctures in time can branch out new trajectories. While transformative learning in our study comes across as the most powerful driver to transcend the rigidness by which we structure the world and what is possible and desirable, it needs to interact with other drivers. Disruptions and times of distorting dilemmas could provide such critical conjunctions, when they coincide with, for example, the evolvement of narratives, economic shifts and socio-technical innovations.

## Supplementary Information

Below is the link to the electronic supplementary material.

Supplementary file1 (DOCX 18 KB)
