# Supplementary material for: Drivers of sustainability transformations: leverage points, contexts and conjunctures
Source: Sustain Sci. 2021 Apr 27;16(3):889–900. doi: 10.1007/s11625-021-00957-4 (PMC8075710; doi:10.1007/s11625-021-00957-4)
Supplement: Supplementary file 1 — Supplementary file1 (DOCX 18 KB) [file 11625_2021_957_MOESM1_ESM.docx]

Supplementary Material

*Drivers of sustainability transformations: leverage points, contexts and conjunctures*

Björn-Ola Linnér and Victoria Wibeck, Department of Thematic Studies – Environmental Change, Linköping University

**Materials and Methods**

For our cross-country study of societal transformations towards sustainability, we conducted sense-making analysis of four types of data: research literature, policy documents, international media texts, and focus group interviews. Our sense-making analyses are inspired by dialogical communication theory (e.g. Bakhtin 1986; Linell 1998, 2009; Marková et al. 2007), which means that sense-making is formed through processes of dialogues between actors, arguments and ideas forging our perceptions, interpretations and viewpoints towards particular phenomena, our existence and the world. To this end, we analyse linguistic resources that are used to make sense of and communicate often multifaceted, unacquainted and/or disputed concepts and phenomena. We pay particular attention to analogies (Marková et al. 2007), metaphors (Lakoff and Johnson 1980), framing (Entman 1993; Goffman 1974) and narratives (Harré et al. 1999).

We selected the analyzed peer-reviewed scholarly literature through searching the Scopus database using the search string TITLE-ABS-KEY ((societ* W/15 transform*) AND NOT (©PRE/10 societ*)) on the titles, abstracts and key words of articles, reviews, book chapters and books. We searched publications in English up to 2016, wich gave in total 6,549 sources. They were analysed through 1) charting their occurrences over time; 2) sorting the publications according to subject areas and geographical locus through the Scopus’ analysis functions; 3) identifying the papers’ most frequently used terms over three distinct periods (1970-1999; 2000-2009; 2010-2016) through the VOSviewer visualization software.

To analyse variations in policy positions between countries, we analysed all Intended and Decided Nationally Determined Contributions (NDCs) to the Paris Agreement as well as Voluntary National Reviews (VNRs) of the UN Sustainable Development Goals. We included all NDCs and VNRs that were submitted to the respective UN bodies by 2018. The 162 NDCs represent 190 parties. Where the NDCs mentioned transformations, we also included the documents to which they referred. 65 VNRs were submitted in 2016-2017. We used the NVivo software to code and sort the NDCs and the VNRs according to which goals, types of transformative actions and actors that were addressed. In addition, we analysed  background documents to which the NDCs refer when discussing transformation, as well as the UN 2030 Agenda and background reports (UN 2013) and the European Green Deal.

We used the Retriever database to identify international English-language media texts addressing various instances of sustainability transformations. We employed three diverse search strings: 1) We searched for (transformation* AND sustainab*) to attain an overview of how media covered transformation towards sustainability; 2) To identify social and society types of transformations toward sustainability specifically, in contrast to the wider varieties of transformations (e.g. personal) we searched for (‘soci* transformation*’ AND sustainab*); 3) lastly, to narrow down the search to identify texts for a deeper analysis of societal transformations toward sustainability, we narrowed the search to (‘societal transformation*’AND sustainab*).

In collaboration with colleagues in five countries we performed focus group interviews with lay people in culturally, politically and economically diverse sites with different environmental and social challenges, where the transformation concept had been explicitly grappled with in official policy documents: Praia in Cabo Verde, Guangzhou in China, the city of Nadi and a village in the Yasawas in Fiji, the Östergötland region in Sweden, and Boulder, Colorado in the USA. In each of these sites four focus groups were recruited, amounting to a total of 20 groups with 136 participants. To facilitate for the participants to share their experiences, most of the focus groups were homogenous in at least one key aspect (e.g., place of residence or age), while we included differences between the groups to encourage a broad span of opinions to come across in the study. The details of this study are reported in Wibeck et al (2019).

**References**

Bakhtin MM (1986) Speech Genres and Other Late Essays. University of Texas Press, Austin, TX.

Entman RM (1993) Framing: toward clarification of a fractured paradigm. Journal of Communication 43:51-58.

Goffman E (1974) Frame Analysis: An Essay on the Organization of Experience. Harvard University Press, Cambridge, MA.

Harré R, Brockmeier J, Mühlhäusler P (1999). Greenspeak: A Study of Environmental Discourse. Sage, Thousand Oaks, CA.

Lakoff G, Johnson M (1980) Metaphors We Live By. Chicago University Press, Chicago, IL.

Linell P (1998) Approaching Dialogue: Talk, Interaction and Contexts in Dialogical Perspectives. John Benjamins Publishing, Amsterdam.

Linell P (2009) Rethinking Language, Mind, and World Dialogically: Interactional and Contextual Theories of Human Sense Making. Information Age Publishing, Charlotte, NC.

Marková I, Linell P, Grossen M, Salazar Orvig A (2007) Dialogue in Focus Groups: Exploring in Socially Shared Knowledge. Equinox Publishing, London.

UN (2013). A New Global Partnership: Eradicate Poverty and Transform Economies through Sustainable Development: The Report of the High-Level Panel of Eminent Persons on the Post-2015 Development Agenda. United Nations, New York.

Wibeck, V., Linnér, B-O., Alves, M., Asplund, T., Bohman, A., Boykoff, M., Feetham, P. M., Huang, Y., Nascimento, J., Rich, J., Rocha C. Y., Vaccarino, F., Xian, S.  (2019) Stories of transformation: a cross-country focus group study on sustainable development and societal change. Sustainability 11:2427.
